# Saw-tooth cardiomyopathy

**DOI:** 10.1186/1532-429X-11-54

**Published:** 2009-12-16

**Authors:** Periklis A Davlouros, Peter G Danias, Ageliki A Karatza, Maria G Kiaffas, Dimitrios Alexopoulos

**Affiliations:** 1 Cardiology Department, Patras University Hospital, Rion, Greece; 2 Cardiac MR Center, Hygeia Hospital, Maroussi Athens, Greece; 3 Tufts University School of Medicine, Boston, MA, USA; 4 Pediatric Clinic, Patras University Hospital, Rion, Greece; 5 Onasis Cardiothoracic Center, Athens, Greece

## Abstract

We present an unusual case of cardiomyopathy in a two month old male infant with a grade-I systolic murmur. Echocardiographic examination disclosed left ventricular (LV), dysplasia with saw-tooth like inwards myocardial projections extending from the lateral walls towards the LV cavity. There was mild LV systolic dysfunction with apical hypokinesia. Cardiovascular magnetic resonance demonstrated in detail these cross bridging muscular projections originating from the inferior interventricular septum and lateral LV wall, along with areas of hypokinesis at the LV septum and apex in a noncoronary distribution, without any late gadolinium enhancement. We have termed this condition saw-tooth cardiomyopathy because of the very characteristic appearance.

## Case Presentation

**Figure 1:**
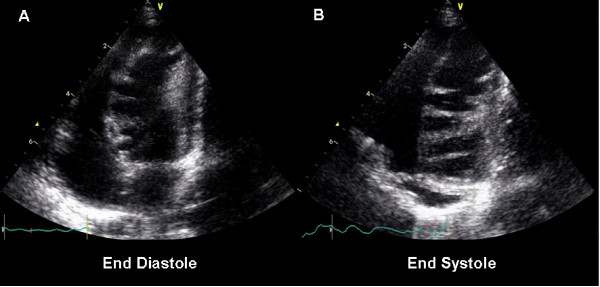
**Echocardiogram**. Apical 4-chamber view with the transducer angulated towards the inferior LV wall in diastole (A) and systole (B). There are numerous echo dense saw tooth like inwards projections from the inferior interventricular septum and lateral LV wall, clearly seen during both systole and diastole. LV apical dyskinesia was also noted.

**Figure 2:**
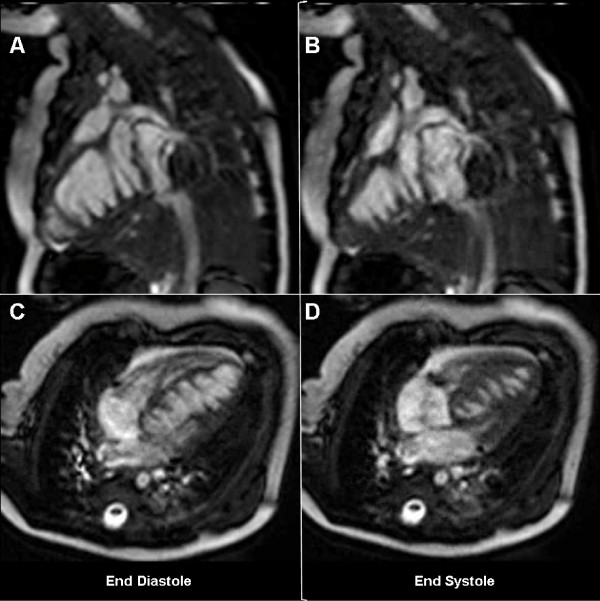
**CMR**. End-diastolic (panels A, C,), and end-systolic (panels B, D) images in the two-chamber and four-chamber orientations, from a free-breathing gradient-echo cine CMR sequence. There are numerous saw-tooth like muscular projections originating from the inferior LV wall, some of them being tethered to the interventricular septum. These myocardial projections have a circular architecture, like successive open rings at the inferior half of the ventricle, spanning the entire heart from the base to the apex.

A two month old asymptomatic male infant was referred to our Congenital Heart Disease Program for assessment of a grade-I systolic murmur discovered during a routine paediatric examination. The mother reported an uncomplicated pregnancy, without any family history of heart disease. There was no history of medication or illegal drug use during pregnancy. Labor was also uncomplicated with an APGAR score of 10. The infant was generally well, with no other clinical signs of heart disease. The electrocardiogram was normal. Brain Natriuretic Peptide (BNP), levels were 352 pg/ml (normal for age < 32.7 pg/ml)[1]. The rest of blood chemistry and thyroid function tests were unremarkable. Initial transthoracic echocardiography demonstrated mild left ventricular (LV) systolic dysfunction, with hypokinesia of the interventricular septum and an aneurysm of the cardiac apex. The striking finding was the existence of numerous echo dense saw tooth like projections from the inferior interventricular septum and LV lateral wall towards the LV cavity. (Figure 1). A patent foramen ovale was also identified with evidence of left to right shunting. The rest of the echocardiographic examination was unremarkable. Despite a slight resemblance with LV non-compaction cardiomyopathy, strict echocardiographic criteria for the diagnosis of this entity in our case did not exist [2]. An alternative hypothesis was that the echo dense projections represented mural thrombi and the possibility of some form of intrauterine LV ischemic insult/accident was postulated. A search in the literature for any similar case was negative. Heart failure treatment with digitalis, carvedilol, furosemide and an ACE-inhibitor was commenced. There was some uncertainty as to whether warfarin should be prescribed for the presumed mural thrombi and the aneurismal apex; therefore the patient was referred for cardiovascular magnetic resonance (CMR) for better anatomic and functional assessment. CMR demonstrated a distorted LV and confirmed the presence of numerous cross bridging muscular projections originating from the inferior wall, inferior interventricular septum and lateral LV walls. Some of these projections seemed to be tethered to the interventricular septum. (Figure 2). There were no findings of non-compaction of either the left or the right ventricles[3]. The LV systolic function was mildly decreased (LV ejection fraction calculated by the Simpson disk-area method 47%) with areas of hypokinesis in a non-coronary distribution at the interventricular septum and apex. Dyskinesia of the latter was clearly demonstrated. Late gadolinium enhancement demonstrated the absence of any myocardial scar. No intracavitary clots were identified. The proximal coronary arteries were visualized at the usual location. The left-to-right shunt was quantified as non-significant. A 24-hour Holter recording did not disclose any arrhythmias. Six months later the patient was well thriving and asymptomatic. An echocardiographic examination demonstrated the same morphologic findings with preservation of the LV systolic function. BNP levels were 89 pg/ml. Furosemide and digitalis were stopped and the child is being followed-up.

The incidence of heart muscle disease induced new-onset heart failure in the absence of congenital heart disease in children, has been recently reported in a nationwide multicenter study from UK and Ireland to be 87 per 100,000 population less than 16 years[4]. The median age at presentation was 1 year in that study and the most common causes of heart failure included dilated cardiomyopathy, probable myocarditis, occult arrhythmia, anthracycline toxicity, metabolic disease and LV non-compaction. Our case constitutes the first report of echocardiographic and CMR of a form of heart muscle disease accompanied by LV systolic dysfunction, characterized by formation of successive open muscular rings at the inferior half of the LV. The relationship of the latter with the reduced LV systolic function-if any-remains obscure; however a presumably deranged myofibril organization pattern might link gross morphology with altered systolic function.

## Conclusion

We present a form of LV dysplasia of the newborn with unique morphologic features, which we have termed saw-tooth cardiomyopathy because of the very characteristic appearance.

## Consent

Written informed consent was obtained from the mother of the infant for publication of this case report and accompanying images. A copy of the written consent is available for review by the Editor-in-Chief of this journal.

## Competing interests

The authors declare that they have no competing interests.

## Authors' contributions

PDavlouros, AK and MK performed independent echocardiograms, interpreted and edited the images and discussed the differential diagnosis of this rare case. PDanias performed the CMR study and interpreted the corresponding images. PDavlouros and PDanias drafted the manuscript. DA helped to write and rewrote the manuscript.
